# Glycine Release Is Upregulated by Metabotropic Glutamate Receptor 1 in Mouse Hippocampus

**DOI:** 10.3390/biomedicines13123106

**Published:** 2025-12-17

**Authors:** Luca Raiteri, Maria Cerminara, Aldamaria Puliti, Anna Pittaluga

**Affiliations:** 1 Department of Pharmacy (DIFAR), Pharmacology and Toxicology Section, University of Genoa, Viale Cembrano 4, 16148 Genoa, Italy; annamaria.pittaluga@unige.it; 2 Medical Genetics Unit, IRCCS Istituto Giannina Gaslini, 16147 Genoa, Italy; mariacerminara@gaslini.org (M.C.); aldamaria.puliti@unige.it (A.P.); 3 Department of Neurosciences, Rehabilitation, Ophthalmology, Genetics, Maternal and Child Health (DiNOGMI), University of Genoa, Largo Paolo Daneo, 16132 Genoa, Italy; 4 IRCCS Ospedale Policlinico San Martino, Largo Rosanna Benzi 10, 16132 Genoa, Italy

**Keywords:** glycine, release, hippocampus, metabotropic glutamate receptors, mGlu1, synaptosomes

## Abstract

**Background/Objectives**: The neurotransmitter glycine is involved in several physiological and pathological conditions in the Central Nervous System. Different biological structures, including glycine receptors and transporters, are under study as targets for potential drugs acting against serious neurological and psychiatric disorders. The regulation of glycine release from nerve terminals is only partially understood. We report here preliminary evidence of the modulation of glycine release through presynaptic metabotropic glutamate receptors 1 (mGlu1) from glycinergic nerve terminals in mouse hippocampi. **Methods**: Purified mouse hippocampal synaptosomes labeled with [^3^H]glycine were used to study glycine release under superfusion conditions. **Results**: The group I metabotropic glutamate receptor agonist 3,5-DHPG potentiated depolarization-evoked [^3^H]glycine release from hippocampal synaptosomes, an effect strongly counteracted by the selective mGlu1 antagonist LY 367385. 3,5-DHPG failed to increase [^3^H]glycine release in *Grm1^crv4/crv4^* mice, a mouse model lacking mGlu1. Although further research is needed to clarify these mechanisms, data suggest that glycine-releasing hippocampal nerve terminals are endowed with presynaptic mGlu1 receptors whose activation potentiates glycine release. **Conclusions**: Considering that in the hippocampus, glycine is relevant as a co-agonist of glutamate at NMDA receptors and that mGlu1 receptor ligands are under study as potential drugs, we propose that the possible effects of these agents on the release of glycine should be considered when studying these compounds.

## 1. Introduction

Glycine (Gly) exerts a dual role in the Central Nervous System (CNS): it is a major inhibitory neurotransmitter (NT) acting at ionotropic Gly receptors [1], and it also participates in excitatory neurotransmission because it is a co-agonist of Glutamate (Glu) at NMDA receptors (NMDARs) [2], and it interacts with other recently discovered excitatory receptors [3]. Knowledge of Gly-mediated neurotransmission has accumulated over the last 25 years (see, for instance, [4,5,6,7,8,9]). Regulation of its release from glycinergic nerve terminals also has been studied [10,11,12,13,14] although it is only partially understood. Given its involvement in relevant CNS physiopathological conditions, Gly-mediated neurotransmission has raised interest, as well as because some related biological structures, including Gly receptors and transporters, are potential targets for novel drugs [15,16,17,18,19,20].

Modulation of Gly release can also occur through presynaptic metabotropic glutamate receptors (mGluRs) [13]. Generally, mGluRs are involved in many physiopathological conditions in the CNS, including presynaptic modulation of NT release; studies related to such receptors are abundant, including reviews: see [21,22,23]. Modulators of group I mGluRs are receiving attention as potential therapeutic agents against CNS pathologies like schizophrenia (SCZ) [22,24] and neurodegenerative disorders [23,25,26].

The objective of the present study is to determine whether presynaptic metabotropic glutamate receptors 1 (mGlu1) modulate Gly release in the hippocampus. Our results suggest the possible existence of presynaptic mGlu1 on Gly-releasing nerve terminals and their stimulatory effect on the release of Gly from isolated nerve terminals (synaptosomes) purified from mouse hippocampi. Considering that ligands of mGlu1 are studied as potential therapeutic drugs and that dysfunctions related to NT Gly are involved in critical CNS disorders, we propose that the possible effects of such agents on the release of Gly should be considered.

## 2. Materials and Methods

### 2.1. Animals

Adult “Swiss” mice (weighing 20–25 g; Charles River, Calco, Italy) were used. Mice were housed in the animal facility of the Department of Pharmacy, Section of Pharmacology and Toxicology, University of Genoa (authorization for animal utilization n. 484, 8 June 2004). Animals were housed at constant temperature (22 ± 1 °C) and relative humidity (50%) under a regular light–dark schedule (light from 7.00 a.m. to 7.00 p.m.). Food and water were freely available. Animal care and experimental procedures complied with European legislation (Directive of 22 September 2010, no. 2010/63/EU), the “ARRIVE” guidelines, and Italian legislation. All efforts were made to minimize animal suffering and to use only the number of animals necessary to produce reliable results.

In a set of experiments, mice lacking the mGlu1 receptor, *Grm1^crv4/crv4^* mice [27], were used. *Grm1^crv4^* mice carry a recessive loss-of-function mutation (*crv4*) in the *Grm1* gene that codes for the mGlu1 receptor. *Crv4* is a spontaneous mutation that occurs in the BALB/c/Pas inbred strain and consists of a retrotransposon long terminal repeat (LTR) fragment insertion. This insertion disrupts the splicing of *Grm1*, resulting in the absence of the protein [27]. Affected (*Grm1^crv4/crv4^*) and control (*Grm1^+/+^*) mice were obtained from the animal facility of the IRCCS Ospedale Policlinico San Martino (Genoa, Italy), where they were maintained with the same genetic background by intercrossing *Grm1^+/crv4^* mice. The genotype of the *Grm1^crv4^* mice was identified by PCR using specific primers, as already reported [28]. Briefly, DNA was extracted from the mice’s tails and amplified by polymerase chain reaction (PCR) using 2 pairs of primers. The first one (5′-GAGTGTTCACTAGTTCACCCAAGA-3′ and 5′-TCAGGCAACAATAAGGCAAG-3′) flanked the insertion and amplified a product of 688 and 498 bp for the *crv4* mutant and the wild type alleles, respectively. The second pair of primers (5′-TGTCAGGGATGAACTGAAAGAA-3′and 5′-GCAGCTCAATTCCCAACAAT-3′) amplified a genomic fragment of 250 bp specific to the LTR insertion of the mutated mGlu1 receptor gene.

### 2.2. Preparation of Synaptosomes

Animals were sacrificed by cervical dislocation. The whole hippocampi were removed from the brain as quickly as possible and transferred into an ice-cold physiological solution (standard medium) with the following composition (mM): NaCl, 140; KCl, 3; MgSO_4_, 1.2; CaCl_2_, 1.2; NaH_2_PO_4_, 1.2; NaHCO_3_, 5; glucose, 10; HEPES, 10; pH adjusted to 7.4 with NaOH. The tissue was then transferred and homogenized in a glass–Teflon tissue grinder with a clearance of 0.25 mm and containing 10 volumes (100 mg/1 mL) of 0.32 M sucrose buffered at pH 7.4 with Tris-HCl, maintained at 0–4 °C. Tissue was homogenized by performing 12 up–down strokes at 900 rpm in about 1 min. To obtain purified synaptosomes [29,30] the homogenate was first centrifuged (5 min, 1000× *g* at 4 °C) to remove nuclei and debris, and the supernatant was gently stratified on a discontinuous Percoll^®^ (Sigma Chemical Co., St. Louis, MO, USA) gradient (2, 6, 10 and 20%, *v*/*v* in Tris-buffered sucrose) obtained as follows. Each gradient was prepared at least 12 h before use by stratifying 3 mL of a 10% (*v*/*v*) solution of Percoll^®^ in Tris-buffered sucrose upon 3 mL of a solution of 20% (*v*/*v*) Percoll^®^ in Tris-buffered sucrose, pH = 7.4; subsequently, 3 mL of a 6% and then 3 mL of a 2% solution of Percoll^®^ in Tris-buffered sucrose were stratified. Stratification of the solutions was performed with a peristaltic pump (flow rate: 1 mL/min). After gradient preparation, each gradient tube was maintained at 0–4 °C in a steady position until use. A maximum of 3 mL of supernatant per gradient was stratified. Gradients were then centrifuged at 33,500× *g* for 5 min (0–4 °C). Each layer between 10% and 20% Percoll^®^ (synaptosomal fraction) was collected with a pipette, washed by centrifugation with ice-cold standard medium, and finally resuspended in room-temperature standard medium and immediately used (see Section 2.3). For further details, see [5]. Protein was determined according to Bradford [31] using bovine serum albumin as a standard. All the reagents were of laboratory grade.

### 2.3. Neurotransmitter Release Experiments

Synaptosomes were incubated at 37 °C for 15 min with [^3^H]Gly (0.3 µM; Perkin Elmer, Boston, MA, USA), in the presence of 0.1 µM of the selective Glycine Transporter 1 (GlyT1) blocker N-[(3R)-3-([1,1′-biphenyl]-4-yloxy)-3-(4-fluorophenyl)propyl]-N-methylglycine hydrochloride (NFPS; Tocris Bioscience, Bristol, UK). After incubation, identical portions of the synaptosomal suspension (about 20 μg protein) were distributed as monolayers on microporous filters placed at the bottom of superfusion chambers in parallel, maintained at 37 °C (Superfusion System, Ugo Basile, Comerio, Varese, Italy), and superfused with standard medium at a flow rate of 0.5 mL/min [6,13]. After 36 min of superfusion, to allow the system to equilibrate, three superfusate fractions were collected (*t* = 36–39, basal release; *t* = 39–45, depolarization-evoked release; *t* = 45–48, basal release). A 90 s period of depolarization with a physiological medium containing 15 mM KCl (substituting for an equimolar concentration of NaCl) was applied at *t* = 39 min of superfusion. The receptor agonist (s)-3,5-Dihydroxyphenylglycine (3,5-DHPG) was present in superfusion during depolarization. The mGlu1 receptor antagonist (S)-(+)-α-Amino-4-carboxy-2-methylbenzene acetic acid (LY 367385) was added 9 min before depolarization (*t* = 30 min of superfusion). Fractions collected and superfused filters were counted for radioactivity by liquid scintillation counting performed with Packard TRI-CARB 2100 TR Liquid Scintillation Analyzer (Packard Instrument Company, Meriden, CT, USA). All the reagents used were of laboratory grade.

### 2.4. Data Analysis

[^3^H]Gly released in each fraction collected was expressed as a percentage of the radioactivity content of synaptosomes at the start of the respective collection period (fractional rate × 100). The depolarization-evoked NT overflow (per cent overflow) was calculated by subtracting the transmitter content in the fractions corresponding to basal release from the transmitter content in the 6 min fraction collected during and after the depolarization stimulus. The effects of drugs were evaluated by calculating the ratio of the depolarization-evoked neurotransmitter overflow in the presence of the drug under study versus the corresponding value calculated under control conditions.

Statistical analysis of the data was performed through the appropriate comparison tests, as indicated in the legends of figures and table. Data were analyzed by the Tukey–Kramer multiple comparison test, the non-parametric Steel–Dwass test, or, when two means were compared, with the two-tailed Student’s *t* test. Differences between means were considered statistically significant for *p* < 0.05. No data points were excluded from analysis, and no outlier test was performed. The free software Kyplot 6.0 (version 6.0.2; KyensLab Inc., Tokyo, Japan) was used for statistical analysis. Sigma Plot 16.0 software was used for graph drawing.

## 3. Results

**Figure 1:**
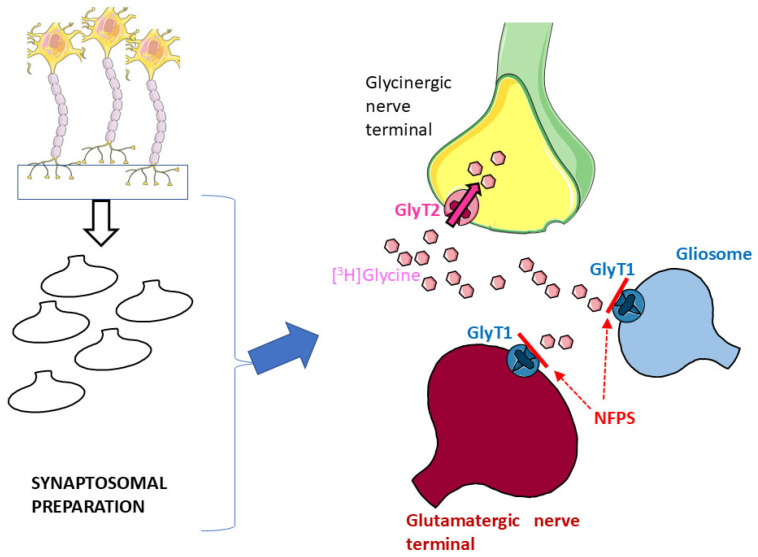
Incubation of hippocampal synaptosomal preparations with [^3^H]Gly in the presence of the selective GlyT1 transporter blocker NFPS should prevent uptake of the radioactive tracer in glial particles (blue) and glutamatergic nerve terminals (red); see Results and references therein for details. Parts of Figure 1 were drawn by using and modifying pictures from Servier Medical Art (Servier; https://smart.servier.com/, accessed on 9 July 2025).

Glycine Transporter 1 (GlyT1) and Glycine Transporter 2 (GlyT2) are the two major high-affinity transporters for Gly. While GlyT1 is largely located on glia and on some populations of glutamatergic nerve terminals [17,32], GlyT2 exhibits quite a selective localization on glycinergic nerve terminals [17,33,34]. Synaptosomal preparations, although purified, can still contain a certain degree of glial particles (gliosomes) originating from astrocytes [5,6] that might be endowed with the “glial” GlyT1. Here, hippocampal synaptosomes were first incubated with [^3^H]Gly in the presence of the selective GlyT1 blocker NFPS to permit [^3^H]Gly to enter through GlyT2 and preferentially label glycinergic nerve terminals ([5,6,11,13]: see Figure 1).

After incubation, synaptosomes were exposed in superfusion to 15 mM KCl, a depolarizing stimulus that causes NT release, essentially, or largely, by external Ca^2+^-dependent exocytosis [5,6,35]; in agreement with several pieces of evidence, tritium release evoked by the depolarization of superfused synaptosomes prelabelled with a tritiated NT with 12–15 mM KCl is an almost completely exocytotic process which reflects the exocytotic release of the endogenous NT under study [5,6,36,37,38,39,40].

**Figure 2:**
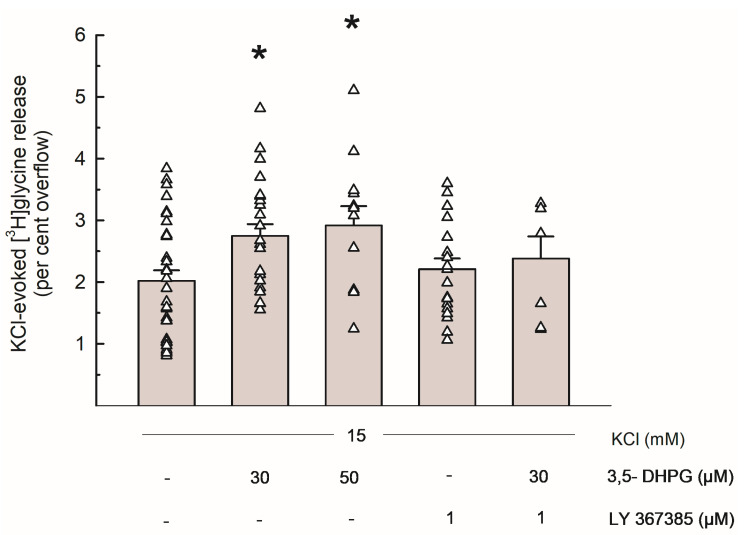
Release of [^3^H]Gly evoked by 15 mM KCl from mouse hippocampal synaptosomes and effects of 3,5-DHPG and LY 367385. Bar data are expressed as per cent overflow and given as means ± SEM of at least 3 independent experiments. Each experiment includes multiple replicates, as indicated by triangles that represent each single data plot. * *p* < 0.05 vs. the respective control value (Tukey–Kramer multiple comparison test). The spontaneous release of [^3^H]Gly in the first fraction collected (basal release) amounted to 3.83 ± 0.26% of the total synaptosomal tritium content (*n* = 16).

Figure 2 shows the [^3^H]Gly release evoked by 15 mM KCl and the effects of the group I mGluRs agonist 3,5-DHPG (30 and 50 µM) and of the selective mGlu1 antagonist LY 367385 (1 µM): when added to the superfusion medium during depolarization, 3,5-DHPG significantly increased the depolarization-evoked [^3^H]Gly overflow at concentrations of 30 and 50 µM. LY 367385 did not exhibit any significant effect on its own, and it markedly reversed the effect of 3,5-DHPG 30 µM.

**Figure 3:**
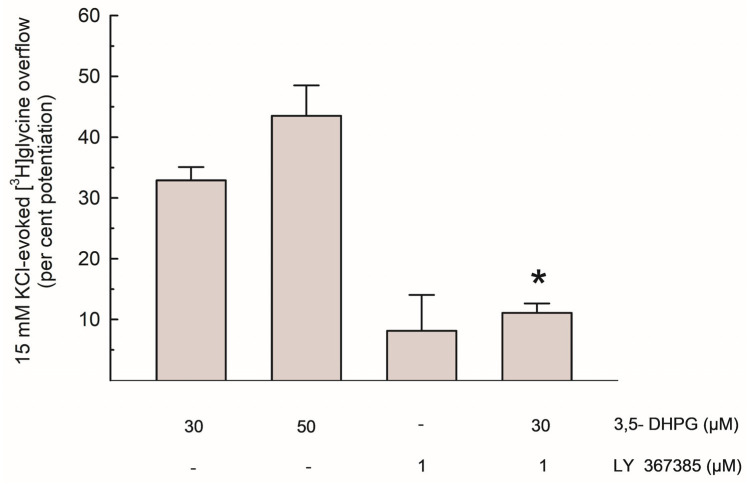
Potentiation of the depolarization-evoked [^3^H]Gly overflow by 3,5-DHPG, alone and in the presence of LY 367385; data are expressed as per cent potentiation of the 15 mM KCl-evoked [^3^H]Gly overflow in control conditions. Results are means ± SEM of at least 3 independent experiments. * *p* < 0.01 vs. the effect of 3,5-DHPG (30 µM) alone (Steel-Dwass non-parametric test).

As shown in Figure 3, the per cent potentiation of the depolarization-evoked [^3^H]Gly release by 3,5-DHPG amounted to about 35% and 45% with 30 µM and 50 µM of the compound, respectively. The effect of 30 µM 3,5-DHPG was reduced by about 65% by LY 367385.

Based on the characteristics of the superfusion technique here exploited (see Section 4.1 and references therein), these results suggest that, in the hippocampus, presynaptic functional mGlu1 exist on Gly-releasing nerve terminals, where they upregulate Gly release, although further research is needed to better understand these mechanisms. Finally, as shown in Table 1, the effect of 3,5-DHPG (50 µM) was tested in synaptosomes obtained from the hippocampi of control and *Grm1^crv4/crv4^* mice, a mouse model lacking mGlu1 [27]. In this set of experiments, 3,5-DHPG potentiated the 15 mM KCl-evoked [^3^H]Gly release by about 43% in control mice, while in *Grm1^crv4/crv4^* mice, the same concentration of 3,5-DHPG failed to increase the depolarization-evoked [^3^H]Gly release (see Table 1).

## 4. Discussion

### 4.1. Methodological Considerations

Purified synaptosomes were chosen as an experimental model, being a suitable in vitro model to study presynaptic events, including NT release [41,42]. Recently, our synaptosomal preparations were characterized at the ultrastructural level by electron microscopy [43,44].

As introduced in Section 3, synaptosomes were labeled with [^3^H]Gly in the presence of the GlyT1 transporter blocker NFPS to perform labeling, mainly through GlyT2, and reduce the labeling of gliosomes. It has to be pointed out that in a few studies, the neuronal GlyT2 transporters were also found on glia [45,46]; therefore, labeling of residual glial particles cannot be completely ruled out. However, we believe that this strategy (labeling of *purified* synaptosomes with a GlyT1 transporter blocker) should at least minimize the labeling of the residual glial particles still present in our synaptosomal preparations.

To study NT release from synaptosomes, we exploited the superfusion technique, a suitable method to study the mechanisms of NT release from nerve terminals, its presynaptic modulation, and the possible interactions among presynaptic receptors controlling NT release [21,35,47,48,49].

### 4.2. Functional Evidence of Presynaptic mGlu1 Able to Increase Gly Release from Hippocampal Gly-Releasing Nerve Terminals

We here investigated the effects of mGlu1 activation on Gly release in the hippocampus because (i) modulators of these receptors are the object of several studies and (ii) Gly and its release are involved in important pathophysiological conditions.

To determine the possible effect of mGlu1 activation on [^3^H]Gly release, we tested the effect of the group I mGluRs agonist 3,5-DHPG, which was added to the superfusion medium at concentrations of 30–50 µM. As shown in Figure 2, the depolarization-evoked [^3^H]Gly release was significantly enhanced by both concentrations of the compound, and the effect of 3,5-DHPG 30 µM was strongly counteracted by the selective mGlu1 antagonist LY 367385 (Figure 2 and Figure 3). Considering the characteristics of the superfusion technique (see Section 4.1 and references therein), we propose that these functional results suggest the existence of presynaptic mGlu1 on Gly-releasing nerve terminals in mouse hippocampi, where their activation increases Gly release. This is compatible with previous evidence of presynaptic, NT release-regulating group I mGluRs behaving as auto- or heteroreceptors (see [21] for a review). Finally, the lack of effect of 3,5-DHPG in *Grm1^crv4/crv4^* mice, a mouse model lacking mGlu1 [27] is compatible with this view (see Table 1). The existence of group I mGluRs able to increase Gly release is also in line with data reported by Saransaari and Oja [7] regarding the upregulation of Gly release by 3,5-DHPG from slices obtained from the brainstem, an area in which Gly is a pivotal inhibitory NT. However, in many CNS areas, including the hippocampus, important roles of Gly also involve its “excitatory” action as a co-agonist of Glu at NMDARs.

With regard to the strength of the effect of 3,5-DHPG, although we did not perform a concentration–response curve, we tend to consider that 50 µM 3,5-DHPG is a concentration able to provide an effect relatively close to the maximal one (see [50]).

### 4.3. Possible Implications of the Upregulation of Gly Release by mGlu1 in the Hippocampus

Novel Positive Allosteric Modulators (PAMs) of Group I mGluRs are indicated as potential antipsychotic drugs: although studies dealing with metabotropic glutamate receptor 5 (mGlu5, the other group I mGluR) seem more advanced, mGlu1 receptors can also play relevant roles; in particular, deleterious mutations in the GRM1 gene, which encodes for mGlu1, have been related to SCZ [51] and subsets of SCZ patients affected by such mutations could benefit from mGlu1 PAMs ([52,53]; reviewed in [22]). mGlu1 PAMs exhibited antipsychotic-like effects in animal models and might play a role against SCZ ([22,24] and references therein), although further studies are required.

Indeed, modulation of the glutamatergic system in different ways is currently a hallmark of research against SCZ, especially with the aim of potentiating families of hypofunctional NMDARs [20,54,55]. Gly is a crucial modulator of the glutamatergic system, since it is an NMDAR co-agonist. Accordingly, one of the approaches in recent research has been represented by the pharmacological blockade of GlyT1, an intervention which is expected to increase Gly availability and therefore enhance hypofunctional NMDARs [15,20]. These considerations are in line with the potential relevance of increased Gly availability at NMDARs in the treatment of certain symptoms of SCZ; in this context, we might speculate that, if group I mGluRs enhancers (including mGlu1 PAMs) can increase Gly release in certain hippocampal regions (similarly to the effect observed here with the agonist 3,5-DHPG) this could be in line with the reported antipsychotic potential of these compounds.

Antagonists/Negative Allosteric Modulators (NAMs) of group I mGluRs can be neuroprotective [56]; interestingly, recent studies suggest potential effects of mGlu1 antagonists/NAMs against neurodegeneration [25,26,50]. We could speculate that, if similar compounds also reduce agonist-induced Gly release (as observed here with LY 367385), this could be linked with neuroprotective effects. NMDARs have both a synaptic and extrasynaptic localization. Non-synaptic/extrasynaptic NMDARs, generally containing the GluN2B subunit, can promote neurotoxicity; Gly is preferentially a co-agonist at extrasynaptic NMDARs, while the D-amino acid D-serine, rather than Gly, is generally considered the main co-agonist at “synaptic” NMDARs [57,58]. As a speculation, we propose that, in case mGlu1 antagonists/NAMs indeed reduce Gly release in vivo, the “neurotoxicity-promoting” effects of extrasynaptic NMDARs might be inhibited. With regard to Gly–Glu reciprocity in ischemic and neurotoxic conditions, this should be partially valid, especially in relation to extrasynaptic NMDARs.

To the best of our knowledge, the concept of Gly modulation could therefore be potentially relevant; in particular, an increase in Gly could be helpful in conditions like SCZ and cognitive impairments, including those caused by ketamine anesthesia. A selective reduction in Gly release onto extrasynaptic NMDARs could perhaps be useful to reduce/prevent neurodegenerative phenomena, but clearly, the extremely complex scenario occurring at the synaptic level implies that it is still difficult to draw conclusions, and therefore, a translational impact is still limited.

## 5. Limitations of the Study

Limitations of the present study include the still-low translational impact of the current observations due to several reasons. First, these investigations were performed with a single in vitro technique, which cannot show direct links between the presynaptic release of Gly and functional responses/changes at the NMDAR level. Second, it is not possible here to understand whether the described effects indeed play relevant pathophysiological roles in vivo, and behavioral evidence is missing. As a consequence, our considerations regarding SCZ and neuroprotection (see Section 4.3) can only be speculative at this stage. Further investigation is required to address these important points.

## 6. Conclusions

To conclude, although with caution (see Section 5), we propose that in the hippocampus, modulators of mGlu1 receptors also regulate Gly release. Confirmation/better understanding of the mechanisms described here requires further investigation. We suggest, however, that the possible effects on Gly release and their implications should be taken into account when studying mGlu1 receptor modulators.

## Figures and Tables

**Table 1:** [^3^H]Gly release evoked by 15 mM KCl and effect of 3,5-DHPG (50 µM) from hippocampal synaptosomes obtained from control and *Grm1^crv4/crv4^* mice.

Control Mice			*Grm1^crv4/crv4^* Mice		
15 mM KCl	15 mM KCl + 3,5-DHPG (50 µM)	% Potentiation	15 mM KCl	15 mM KCl + 3,5-DHPG (50 µM)	% Potentiation
2.13 ± 0.033 (3)	3.05 ± 0.13 (3) *	+43.41	2.18 ± 0.31 (3)	1.90 ± 0.29 (3)	−12.84

Data are expressed as per cent overflow and given as means ± SEM of the number of experiments in parentheses. * *p* < 0.05 vs. the respective control value with the two-tailed Student’s *t*-test, t = −6.756955.

## Data Availability

Data are available on request from the corresponding author.

## References

[1] Lynch J.W. (2009). Native Glycine Receptor Subtypes and Their Physiological Roles. Neuropharmacology.

[2] Johnson J.W., Ascher P. (1987). Glycine Potentiates the NMDA Response in Cultured Mouse Brain Neurons. Nature.

[3] Laboute T., Zucca S., Holcomb M., Patil D.N., Garza C., Wheatley B.A., Roy R.N., Forli S., Martemyanov K.A. (2023). Orphan Receptor GPR158 Serves as a Metabotropic Glycine Receptor: mGlyR. Science.

[4] Geerlings A., Núñez E., López-Corcuera B., Aragón C. (2001). Calcium- and Syntaxin 1-Mediated Trafficking of the Neuronal Glycine Transporter GLYT2. J. Biol. Chem..

[5] Luccini E., Raiteri L. (2007). Mechanisms of [(3)H]Glycine Release from Mouse Spinal Cord Synaptosomes Selectively Labeled through GLYT2 Transporters. J. Neurochem..

[6] Luccini E., Romei C., Raiteri L. (2008). Glycinergic Nerve Endings in Hippocampus and Spinal Cord Release Glycine by Different Mechanisms in Response to Identical Depolarizing Stimuli. J. Neurochem..

[7] Saransaari P., Oja S.S. (2009). Mechanisms of Glycine Release in Mouse Brain Stem Slices. Neurochem. Res..

[8] Harsing L.G., Matyus P. (2013). Mechanisms of Glycine Release, Which Build up Synaptic and Extrasynaptic Glycine Levels: The Role of Synaptic and Non-Synaptic Glycine Transporters. Brain Res. Bull..

[9] Waseem T.V., Fedorovich S.V. (2010). Presynaptic Glycine Receptors Influence Plasma Membrane Potential and Glutamate Release. Neurochem. Res..

[10] Kerchner G.A., Wang G.-D., Qiu C.-S., Huettner J.E., Zhuo M. (2001). Direct Presynaptic Regulation of GABA/Glycine Release by Kainate Receptors in the Dorsal Horn. Neuron.

[11] Romei C., Luccini E., Raiteri M., Raiteri L. (2009). GABA(B) Presynaptic Receptors Modulate Glycine Exocytosis from Mouse Spinal Cord and Hippocampus Glycinergic Nerve Endings. Pharmacol. Res..

[12] Zappettini S., Mura E., Grilli M., Preda S., Salamone A., Olivero G., Govoni S., Marchi M. (2011). Different Presynaptic Nicotinic Receptor Subtypes Modulate in Vivo and in Vitro the Release of Glycine in the Rat Hippocampus. Neurochem. Int..

[13] Romei C., Raiteri M., Raiteri L. (2013). Glycine Release Is Regulated by Metabotropic Glutamate Receptors Sensitive to mGluR2/3 Ligands and Activated by N-Acetylaspartylglutamate (NAAG). Neuropharmacology.

[14] Kalinina N.I., Zaitsev A.V., Vesselkin N.P. (2018). Presynaptic Serotonin 5-HT1B/D Receptor-Mediated Inhibition of Glycinergic Transmission to the Frog Spinal Motoneurons. J. Comp. Physiol. A.

[15] Cioffi C.L., Guzzo P.R. (2016). Inhibitors of Glycine Transporter-1: Potential Therapeutics for the Treatment of CNS Disorders. Curr. Top. Med. Chem..

[16] Söderpalm B., Lidö H.H., Ericson M. (2017). The Glycine Receptor—A Functionally Important Primary Brain Target of Ethanol. Alcohol. Clin. Exp. Res..

[17] Marques B.L., Oliveira-Lima O.C., Carvalho G.A., De Almeida Chiarelli R., Ribeiro R.I., Parreira R.C., Da Madeira Freitas E.M., Resende R.R., Klempin F., Ulrich H. (2020). Neurobiology of Glycine Transporters: From Molecules to Behavior. Neurosci. Biobehav. Rev..

[18] Cioffi C.L. (2021). Inhibition of Glycine Re-Uptake: A Potential Approach for Treating Pain by Augmenting Glycine-Mediated Spinal Neurotransmission and Blunting Central Nociceptive Signaling. Biomolecules.

[19] Gallagher C.I., Ha D.A., Harvey R.J., Vandenberg R.J. (2022). Positive Allosteric Modulators of Glycine Receptors and Their Potential Use in Pain Therapies. Pharmacol. Rev..

[20] Piniella D., Zafra F. (2023). Functional Crosstalk of the Glycine Transporter GlyT1 and NMDA Receptors. Neuropharmacology.

[21] Olivero G., Vergassola M., Cisani F., Roggeri A., Pittaluga A. (2020). Presynaptic Release-Regulating Metabotropic Glutamate Receptors: An Update. Curr. Neuropharmacol..

[22] Dogra S., Conn P.J. (2022). Metabotropic Glutamate Receptors As Emerging Targets for the Treatment of Schizophrenia. Mol. Pharmacol..

[23] Nicoletti F., Di Menna L., Iacovelli L., Orlando R., Zuena A.R., Conn P.J., Dogra S., Joffe M.E. (2023). GPCR Interactions Involving Metabotropic Glutamate Receptors and Their Relevance to the Pathophysiology and Treatment of CNS Disorders. Neuropharmacology.

[24] Fadgyas-Stanculete M., Capatina O.O. (2025). Glutamate-Based Therapeutic Strategies for Schizophrenia: Emerging Approaches Beyond Dopamine. Int. J. Mol. Sci..

[25] Milanese M., Bonifacino T., Torazza C., Provenzano F., Kumar M., Ravera S., Zerbo A.R., Frumento G., Balbi M., Nguyen T.P.N. (2021). Blocking Glutamate mGlu_5_ Receptors with the Negative Allosteric Modulator CTEP Improves Disease Course in SOD1^G93A^ Mouse Model of Amyotrophic Lateral Sclerosis. Br. J. Pharmacol..

[26] Witkin J.M., Pandey K.P., Smith J.L. (2022). Clinical Investigations of Compounds Targeting Metabotropic Glutamate Receptors. Pharmacol. Biochem. Behav..

[27] Conti V., Aghaie A., Cilli M., Martin N., Caridi G., Musante L., Candiano G., Castagna M., Fairen A., Ravazzolo R. (2006). Crv4, a Mouse Model for Human Ataxia Associated with Kyphoscoliosis Caused by an mRNA Splicing Mutation of the Metabotropic Glutamate Receptor 1 (Grm1). Int. J. Mol. Med..

[28] Rossi P.I.A., Musante I., Summa M., Pittaluga A., Emionite L., Ikehata M., Rastaldi M.P., Ravazzolo R., Puliti A. (2013). Compensatory Molecular and Functional Mechanisms in Nervous System of the Grm1crv4 Mouse Lacking the mGlu1 Receptor: A Model for Motor Coordination Deficits. Cereb. Cortex.

[29] Dunkley P.R., Heath J.W., Harrison S.M., Jarvie P.E., Glenfield P.J., Rostas J.A.P. (1988). A Rapid Percoll Gradient Procedure for Isolation of Synaptosomes Directly from an S1 Fraction: Homogeneity and Morphology of Subcellular Fractions. Brain Res..

[30] Nakamura Y., Iga K., Shibata T., Shudo M., Kataoka K. (1993). Glial Plasmalemmal Vesicles: A Subcellular Fraction from Rat Hippocampal Homogenate Distinct from Synaptosomes. Glia.

[31] Bradford M.M. (1976). A Rapid and Sensitive Method for the Quantitation of Microgram Quantities of Protein Utilizing the Principle of Protein-Dye Binding. Anal. Biochem..

[32] Cubelos B., Giménez C., Zafra F. (2005). Localization of the GLYT1 Glycine Transporter at Glutamatergic Synapses in the Rat Brain. Cereb. Cortex.

[33] Poyatos I., Ponce J., Aragón C., Giménez C., Zafra F. (1997). The Glycine Transporter GLYT2 Is a Reliable Marker for Glycine-Immunoreactive Neurons. Brain Res. Mol. Brain Res..

[34] Eulenburg V., Armsen W., Betz H., Gomeza J. (2005). Glycine Transporters: Essential Regulators of Neurotransmission. Trends Biochem. Sci..

[35] Fink K.B., Göthert M. (2007). 5-HT Receptor Regulation of Neurotransmitter Release. Pharmacol. Rev..

[36] Najib A., Pelliccioni P., Gil C., Aguilera J. (1999). Clostridium Neurotoxins Influence Serotonin Uptake and Release Differently in Rat Brain Synaptosomes. J. Neurochem..

[37] Sbrenna S., Marti M., Morari M., Calo’ G., Guerrini R., Beani L., Bianchi C. (2000). Modulation of 5-Hydroxytryptamine Efflux from Rat Cortical Synaptosomes by Opioids and Nociceptin. Br. J. Pharmacol..

[38] Raiteri L., Zappettini S., Milanese M., Fedele E., Raiteri M., Bonanno G. (2007). Mechanisms of Glutamate Release Elicited in Rat Cerebrocortical Nerve Endings by “pathologically” Elevated Extraterminal K+ Concentrations. J. Neurochem..

[39] Cavallero A., Marte A., Fedele E. (2009). L-Aspartate as an Amino Acid Neurotransmitter: Mechanisms of the Depolarization-Induced Release from Cerebrocortical Synaptosomes. J. Neurochem..

[40] Maura G., Gemignani A., Versace P., Martire M., Raiteri M. (1982). Carrier-Mediated and Carrier-Independent Release of Serotonin from Isolated Central Nerve Endings. Neurochem. Int..

[41] Langer S.Z. (2008). Presynaptic Autoreceptors Regulating Transmitter Release. Neurochem. Int..

[42] Raiteri M. (2008). Presynaptic Metabotropic Glutamate and GABAB Receptors. Pharmacology of Neurotransmitter Release.

[43] Cortese K., Gagliani M.C., Raiteri L. (2023). Interactions between Glycine and Glutamate through Activation of Their Transporters in Hippocampal Nerve Terminals. Biomedicines.

[44] Olivero G., Taddeucci A., Vallarino G., Trebesova H., Roggeri A., Gagliani M.C., Cortese K., Grilli M., Pittaluga A. (2024). Complement Tunes Glutamate Release and Supports Synaptic Impairments in an Animal Model of Multiple Sclerosis. Br. J. Pharmacol..

[45] Aroeira R.I., Sebastião A.M., Valente C.A. (2014). GlyT1 and GlyT2 in Brain Astrocytes: Expression, Distribution and Function. Brain Struct. Funct..

[46] Aroeira R.I., Sebastião A.M., Valente C.A. (2015). BDNF, via Truncated TrkB Receptor, Modulates GlyT1 and GlyT2 in Astrocytes. Glia.

[47] Westphalen R.I., Kwak N.-B., Daniels K., Hemmings H.C. (2011). Regional Differences in the Effects of Isoflurane on Neurotransmitter Release. Neuropharmacology.

[48] Popoli M., Yan Z., McEwen B.S., Sanacora G. (2012). The Stressed Synapse: The Impact of Stress and Glucocorticoids on Glutamate Transmission. Nat. Rev. Neurosci..

[49] Pittaluga A. (2021). Presynaptic Release-regulating NMDA Receptors in Isolated Nerve Terminals: A Narrative Review. Br. J. Pharmacol..

[50] Bonifacino T., Rebosio C., Provenzano F., Torazza C., Balbi M., Milanese M., Raiteri L., Usai C., Fedele E., Bonanno G. (2019). Enhanced Function and Overexpression of Metabotropic Glutamate Receptors 1 and 5 in the Spinal Cord of the SOD1G93A Mouse Model of Amyotrophic Lateral Sclerosis during Disease Progression. Int. J. Mol. Sci..

[51] Ayoub M.A., Angelicheva D., Vile D., Chandler D., Morar B., Cavanaugh J.A., Visscher P.M., Jablensky A., Pfleger K.D.G., Kalaydjieva L. (2012). Deleterious GRM1 Mutations in Schizophrenia. PLoS ONE.

[52] Cho H.P., Garcia-Barrantes P.M., Brogan J.T., Hopkins C.R., Niswender C.M., Rodriguez A.L., Venable D.F., Morrison R.D., Bubser M., Daniels J.S. (2014). Chemical Modulation of Mutant mGlu_1_ Receptors Derived from Deleterious *GRM1* Mutations Found in Schizophrenics. ACS Chem. Biol..

[53] Garcia-Barrantes P.M., Cho H.P., Niswender C.M., Byers F.W., Locuson C.W., Blobaum A.L., Xiang Z., Rook J.M., Conn P.J., Lindsley C.W. (2015). Development of Novel, CNS Penetrant Positive Allosteric Modulators for the Metabotropic Glutamate Receptor Subtype 1 (mGlu_1_), Based on an *N.*-(3-Chloro-4-(1,3-Dioxoisoindolin-2-Yl)Phenyl)-3-Methylfuran-2-Carboxamide Scaffold, That Potentiate Wild Type and Mutant mGlu_1_ Receptors Found in Schizophrenics. J. Med. Chem..

[54] De Bartolomeis A., Vellucci L., Austin M.C., De Simone G., Barone A. (2022). Rational and Translational Implications of D-Amino Acids for Treatment-Resistant Schizophrenia: From Neurobiology to the Clinics. Biomolecules.

[55] Javitt D.C. (2023). Cognitive Impairment Associated with Schizophrenia: From Pathophysiology to Treatment. Annu. Rev. Pharmacol. Toxicol..

[56] Nicoletti F., Bruno V., Catania M.V., Battaglia G., Copani A., Barbagallo G., Ceña V., Sanchez-Prieto J., Spano P.F., Pizzi M. (1999). Group-I Metabotropic Glutamate Receptors: Hypotheses to Explain Their Dual Role in Neurotoxicity and Neuroprotection. Neuropharmacology.

[57] Papouin T., Ladépêche L., Ruel J., Sacchi S., Labasque M., Hanini M., Groc L., Pollegioni L., Mothet J.-P., Oliet S.H.R. (2012). Synaptic and Extrasynaptic NMDA Receptors Are Gated by Different Endogenous Coagonists. Cell.

[58] Cunha Xavier Pinto M., Lima I.V.D.A., Pessoa Da Costa F.L., Rosa D.V., Mendes-Goulart V.A., Resende R.R., Romano-Silva M.A., Pinheiro De Oliveira A.C., Gomez M.V., Gomez R.S. (2015). Glycine Transporters Type 1 Inhibitor Promotes Brain Preconditioning against NMDA-Induced Excitotoxicity. Neuropharmacology.

